# Frailty score for elderly patients is associated with short-term clinical outcomes in patients with ST-segment elevated myocardial infarction treated with primary percutaneous coronary intervention

**DOI:** 10.1007/s12471-019-1240-7

**Published:** 2019-02-15

**Authors:** M. P. J. Hermans, D. C. Eindhoven, L. A. M. van Winden, G. J. de Grooth, G. J. Blauw, M. Muller, M. J. Schalij

**Affiliations:** 1 Department of Cardiology, Leiden University Medical Centre, Leiden, The Netherlands; 2 Department of Internal/Geriatric Medicine, Leiden University Medical Centre, Leiden, The Netherlands; 3 Department of Internal/Geriatric Medicine, VU University Medical Centre, Amsterdam, The Netherlands

**Keywords:** ST-elevation myocardial infarction, Frail elderly, Percutaneous coronary intervention

## Abstract

**Objective:**

Consistent with the aging population in the Western world, there is a growing number of elderly patients with ST-segment elevation myocardial infarction (STEMI). Primary percutaneous coronary intervention (PCI) is the recommended reperfusion strategy in elderly patients; risk models to determine which of these patients are prone to have poor clinical outcomes are, however, essential. The purpose of this study was to assess the association between frailty and short-term mortality and PCI-related serious adverse events (SAE) in elderly patients.

**Methods:**

All STEMI patients (aged ≥70 years) treated with primary PCI in 2013–2015 at the Leiden University Medical Centre were assessed. The Safety Management Programme (VMS) score was used to identify frail elderly patients. The primary endpoint was 30-day all-cause mortality; the secondary endpoint included 30-day clinical death, target vessel failure, major bleeding, contrast induced kidney insufficiency and stroke.

**Results:**

A total of 206 patients were included (79 ± 6.4 years, 119 [58%] male). The VMS score was ≥1 in 28% of all cases. Primary and secondary endpoint rates were 5 and 23% respectively. VMS score ≥1 was an independent predictor for both 30-day mortality (odds ratio [OR] 9.6 [95% confidence interval, CI 1.6–56.9] *p*-value = 0.013) and 30-day SAE (OR 2.9 [95% CI 1.1–7.9] *p*-value = 0.038).

**Conclusions:**

VMS score for frailty is independently associated with short-term mortality and PCI-related SAE in elderly patients with STEMI treated with primary PCI. These results suggest that frailty in elderly patients is an important feature to measure and to be taken into account when developing risk models.

**Electronic supplementary material:**

The online version of this article (10.1007/s12471-019-1240-7) contains supplementary material, which is available to authorized users.

## What’s new?

- In patients older than 70 years treated with primary percutaneous coronary intervention (PCI) for ST-segment elevation myocardial infarction, a positive frailty score was associated with short-term mortality and short-term PCI-related serious adverse events, most commonly 30-day mortality and major bleeding.
- These findings were independent of age and clinical characteristics; patients with at least one sign of frailty had a nearly 10 times higher risk of 30-day mortality.
- The results of this study emphasise that frailty in the elderly should be taken into account when developing risk models.

## Introduction

Consistent with the aging population in the Western world, there is a growing number of elderly patients with ST-segment elevation myocardial infarction (STEMI) [1–4]. The literature shows that older STEMI patients are at high risk for short-term mortality [5]. Primary percutaneous coronary intervention (PCI) is the recommended treatment, based on results of pooled analysis of small clinical trials in the elderly [6–10].

There is, however, large heterogeneity in the elderly: it is estimated that 25–50% of the population >85 years of age is frail and have impaired prognosis in comparison with the 50–75% that are still vital [11, 12]. Frailty has been described as a condition of vulnerability due to poor recovery of homoeostasis after an event and is a result of cumulative decline in several physiological systems [13]. Cognitive decline, weight loss, self-reported exhaustion, low energy expenditure, slow gait speed and weak grip strength are the phenotype model indicators of frailty. Despite the predictive value of frailty scores for short-term adverse events in the elderly, this factor remains underrepresented in risk models, due to a lack of validated frailty scores. As part of a nationwide Safety Management Programme (*VeiligheidsManagementSysteem* = VMS) the systematic screening of elderly persons regarding undernutrition, activities of daily living (ADL), limitations, falls and cognitive impairment/delirium has been implemented in Dutch Hospitals since 2012 [14]. This screening tool enables the detection of patients at risk in an early stage of hospitalisation, in order to initiate additional measures to prevent adverse events.

Based on clinical experience, we hypothesised that frailty might be a better predictor of adverse clinical events than age. Therefore, we conducted a cohort study to assess whether frailty, assessed by the VMS screening tool, is associated with short-term mortality and PCI-related events in older STEMI patients treated with primary PCI.

## Methods

Leiden University Medical Centre serves a population of 750,000 inhabitants, performing 24 h/7 days a week primary PCI procedures. The study cohort consisted of consecutive STEMI patients aged ≥70 years treated with primary PCI between 2013 and 2015. STEMI was defined as ongoing chest pain (>30 min), accompanied by ST elevation (≥0.2 mV in ≥2 leads in V1–V3 or ≥0.1 mV in other leads) or presumed new left bundle branch block and a typical rise and fall of cardiac biomarkers. At inclusion, demographics, risk factors and clinical characteristics were collected.

Endpoints of this study were 30-day all-cause mortality and the combined endpoint of 30-day clinical outcomes, including death, target vessel failure, major bleeding (transfusion and/or intervention required), contrast-induced kidney insufficiency and stroke. Information on all-cause mortality was obtained from the Dutch Municipality Records registry. Clinical follow-up data on serious adverse events (SAE) including myocardial infarction, revascularisation and stroke were collected during the 30-day outpatient clinic visits. In cases of out-of-hospital cardiac arrest, only patients with return of spontaneous circulation at the moment of arrival at the catheterisation laboratory were included. For patients who lived in another region, only mortality data were available. Therefore, they were excluded from the combined endpoint (clinical outcome at 30 days) analysis. Based on short-term event rates of previous studies that included older patients with STEMI (ca. 10%), it was estimated that a study size of approximately 200 patients would be sufficient [15]. The institutional ethical committee approved this evaluation and waived the need for patient written informed consent for retrospective analysis of clinically collected data.

During the study, all patients were treated according to the institutional STEMI protocol, based on guidelines of the European Society of Cardiology, American College of Cardiology and the American Heart Association [16, 17]. The pre-hospital protocol included diagnosis by field triage by 12-lead electrocardiogram and in-ambulance treatment with a loading dose of clopidogrel, acetylsalicylic acid, heparin, and intravenous glycoprotein IIb/IIIa inhibitors. Patients were directly transferred to the catheterisation laboratory as soon as the procedure could start. Primary PCI was performed according to current guidelines. If tolerated, patients received beta-blockers, angiotensin I converting enzyme (ACE) inhibitors and statins within 24 h. Additionally, patients were prescribed dual antiplatelet therapy, consisting of acetylsalicylic acid 100 mg daily for life and clopidogrel 75 mg daily for 12 months. Patients with an indication for oral anti-coagulants were prescribed coumarin derivatives instead of acetylsalicylic acid.

### VMS score

All patients were scored with the screening tool of the Dutch VMS programme at admission [14]. This screening tool consisted of 12 questions on activities of ADL, falls, undernutrition and cognitive impairment/delirium. For functional status, the six-item Katz Index on Independence in Activities in Daily Living was used, consisting of questions on help with bathing, dressing, toileting, transferring from bed to a chair, eating and the use of incontinence materials [18]. Patients were considered to be at risk for falls if they had experienced a fall in the last 6 months before admission. The risk for undernutrition was assessed with questions concerning unintentional weight loss, decreased appetite and the use of supplemental drinks or tube feeding. If patients had memory problems, needed help with self-care and had previously experienced confusion, they were considered to be at risk for developing delirium. A total VMS score was calculated by adding up the patients’ dichotomised screening results on the four domains, resulting in a score from 0 to 12.

### Statistical analysis

Patients with a VMS score ≥1 were considered frail, since a previous validation study for the VMS score showed that a score ≥1 was associated with impaired clinical outcomes. Normally distributed continuous variables were reported as mean and standard deviation, and compared with Student’s *t*-test for means. Skewed distributed continuous variables were reported as median and interquartile range, and compared with a non-parametric test. Categorical variables were reported as number and percentage, and compared with the Pearson’s chi-square test. There were no missing data regarding 30-day mortality and clinical outcomes. Event-free survival was analysed with Kaplan-Meier estimates and compared between groups with the log-rank test. Logistic regression was performed to assess the association between VMS score and primary and secondary outcome measures. Variables were used in multivariate models if *p*-values were <0.05. All statistical tests were performed with SPSS software (Version 22.0, SPSS IBM Corp., Armonk, NY, USA). *p*-Values <0.05 assessed by two-sided tests were considered to be statistically significant.

## Results

### Study population

A total of 206 patients with STEMI were included for analysis. No follow-up data on PCI-related SAE were obtained from patients with outpatient clinic visits in other regional hospitals (*n* = 63). No differences in baseline characteristics were observed between the group with and without follow-up, except for age (Supplementary file, Table 1). Mean age was 79 ± 6.4 years; 58% of the patients were male. Hypertension was the most common risk factor, with an incidence of 63%; 15% had previously suffered from myocardial infarction. The VMS score was ≥1 in 28% of all cases (Tab. 1).

**Table 1:** Baseline characteristics

	total, *n* (%)	VMS <1, *n* (%)	VMS ≥1, *n* (%)	valid cases
number	206	149	57	
*demographic*				
– age, mean (SD), years	79 (6.4)	77 (5.8)	82 (6.9)	206
– female	87 (42)	54 (36)	33 (58)	206
*risk factors*				
– treated hypertension^a^	124 (63)	90 (63)	34 (61)	198
– treated hyperlipidaemia^b^	48 (24)	35 (25)	13 (24)	197
– diabetes	31 (15)	13 (9)	18 (32)	201
– current smoker	43 (23)	32 (24)	11 (20)	190
– family history of CVD	44 (24)	33 (25)	11 (20)	187
*comorbid conditions*				
– history of cancer	31 (15)	14 (9)	17 (30)	205
– history of peripheral vascular disease	17 (8)	11 (7)	6 (11)	205
– history of cerebrovascular disease	27 (13)	14 (9)	13 (23)	204
– previous myocardial infarction	31 (15)	20 (13)	11 (19)	206
– previous PCI	23 (11)	15 (10)	8 (14)	205
– previous CABG	9 (4)	6 (4)	3 (5)	206
*clinical characteristics*				
– out-of-hospital cardiac arrest	9 (4)	6 (4)	3 (5)	206
– anterior infarction	70 (34)	49 (33)	21 (37)	206
– abciximab administration	151 (74)	115 (79)	36 (63)	203

*CABG* coronary artery bypass graft surgery, *CVD* cardiovascular disease, *PCI* primary percutaneous coronary intervention, *SD* standard deviation, *VMS* Safety Management Programme

^a^Defined as systolic blood pressure ≥140 mm Hg and/or diastolic blood pressure ≥90 mm Hg and/or the use of antihypertensive medication

^b^Serum total cholesterol ≥6.0 mmol/l and/or serum triglycerides ≥2.2 mmol/l or treatment with lipid-lowering drugs

### Association between VMS and mortality

**Fig. 1:**
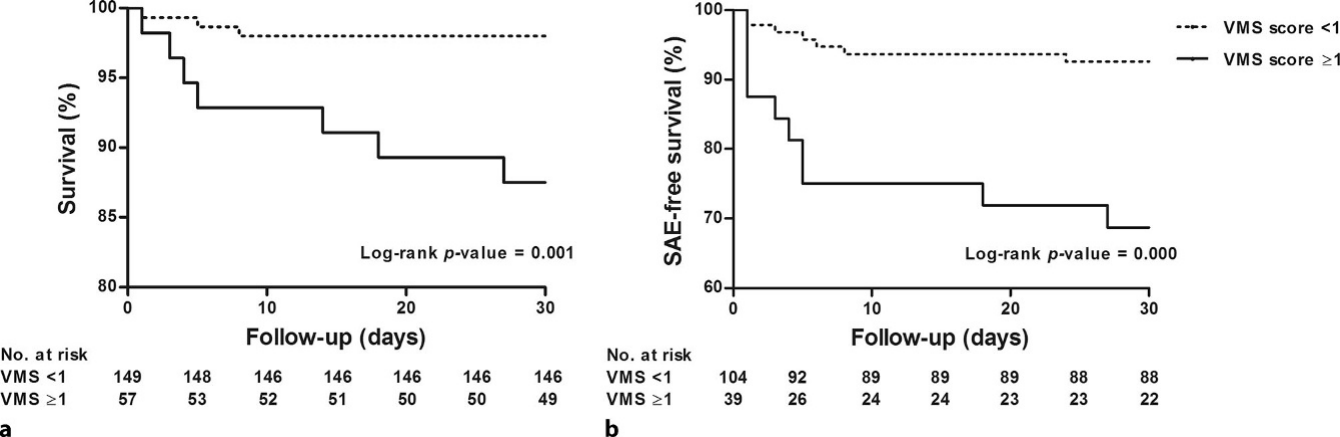
Survival and serious adverse event (*SAE*)-free survival. Kaplan-Meier cumulative 30-day survival (**a**) and SAE-free survival (**b**)

**Fig. 2:**
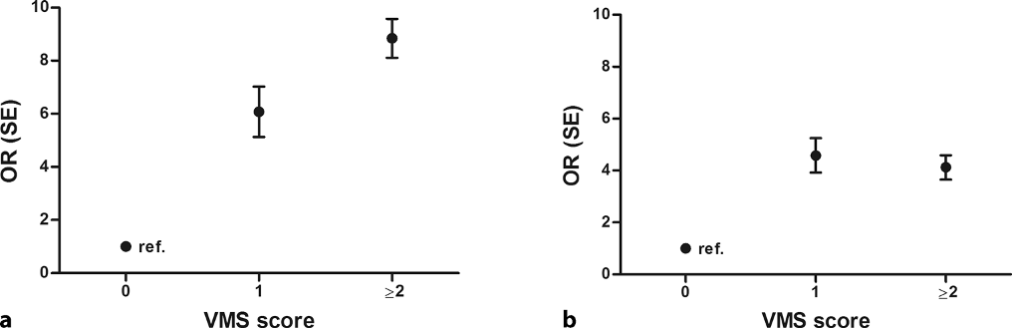
Odds ratios (*OR*) for Safety Management Programme (*VMS*) score. OR and 30-day mortality (**a**) and 30-day serious adverse events (*SAE*) (**b**). (*PCI* percutaneous coronary intervention, *SE* standard error)

The 30-day all-cause mortality rate was 5% (*n* = 11). Kaplan-Meier analysis showed a significant difference between survival of patients with a VMS score <1 and patients with a VMS score ≥1 (log-rank *p*-value = 0.001) (Fig. 1). Logistic regression showed that age (odds ratio [OR] 1.1 [95% confidence interval, CI 1.0–1.2]), diabetes (OR 6.3 [95% CI 1.7–23.4]), history of peripheral vascular disease (OR 8.0 [95% CI 2.1–30.7]), history of cerebrovascular disease (OR 4.2 [95% CI 1.1–15.5]) and VMS score ≥1 (OR 7.9 [95% CI 2.0–31.1]) were associated with 30-day mortality. A history of peripheral vascular disease (OR 8.7 [95% CI 1.4–52.3]) and VMS score ≥1 (OR 9.6 [95% CI 1.6–56.9]) were independently associated with higher 30-day mortality (Tab. 2). The OR for 30-day mortality increased with VMS score (VMS score ≥1 OR 6.1 [95% CI 0.9–39.2]; VMS score ≥2 OR 8.8 [95% CI 2.1–37.2]) (Fig. 2).

**Table 2:** Univariable and multivariable logistic regression analysis of 30-day mortality

	univariable logistic regression		multivariable logistic regression	
	OR (95% CI)	*p*-value	OR (95% CI)	*p*-value
*demographic*				
– age, years	1.1 (1.0–1.2)	0.020	1.0 (0.9–1.2)	0.734
– female	1.1 (0.3–3.9)	0.824	–	–
*risk factors*				
– treated hypertension^a^	1.4 (0.4–5.7)	0.622	–	–
– treated hyperlipidaemia^b^	0.8 (0.2–3.7)	0.742	–	–
– diabetes	6.3 (1.7–23.4)	0.006	2.5 (0.5–13.2)	0.266
– current smoker	0.8 (0.2–4.1)	0.838	–	–
– family history of CVD	0.8 (0.2–3.9)	0.787	–	–
*comorbid conditions*				
– history of cancer	0.5 (0.1–4.4)	0.572	–	–
– history of peripheral vascular disease	8.0 (2.1–30.7)	0.003	8.7 (1.4–52.3)	0.019
– history of cerebrovascular disease	4.2 (1.1–15.5)	0.030	2.0 (0.4–10.4)	0.422
– previous myocardial infarction	2.2 (0.6–8.9)	0.255	–	–
– previous PCI	1.8 (0.4–9.0)	0.458	–	–
– previous CABG	0.0 (0.0–~)	0.999	–	–
*clinical characteristics*				
– out-of-hospital cardiac arrest	2.3 (0.3–20.6)	0.444	–	–
– anterior infarction	1.1 (0.3–4.0)	0.864	–	–
– abciximab administration	0.3 (0.1–1.2)	0.083	–	–
– VMS score ≥1	7.9 (2.0–31.1)	0.003	9.6 (1.6–56.9)	0.013

*CABG* coronary artery bypass graft surgery, *CI* confidence interval, *CVD* cardiovascular disease, *OR* odds ratio, *PCI* primary percutaneous coronary intervention, *VMS* Safety Management Programme

^a^Defined as systolic blood pressure ≥140 mm Hg and/or diastolic blood pressure ≥90 mm Hg and/or the use of antihypertensive medication

^b^Serum total cholesterol ≥6.0 mmol/l and/or serum triglycerides ≥2.2 mmol/l or treatment with lipid-lowering drugs

### Association between VMS and SAE

Follow-up data regarding 30-day PCI-related SAE were available in 143 patients. In total, 33 (23%) patients had PCI-related SAE: 13 (9%) patients suffered from major bleeding, 10 (7%) patients died, 7 (5%) patients had de novo kidney insufficiency, 2 (1%) target vessel failure and 1 (1%) stroke. A significant difference between PCI-related SAE-free survival of patients with a VMS score <1 and patients with a VMS score ≥1 (log-rank *p*-value <0.001) was shown with Kaplan-Meier analysis (Fig. 1) After adjusting for baseline characteristics age (OR 1.1 [95% CI 1.0–1.2]), out of hospital cardiac arrest (OR 6.7 [95% CI 1.0–43.1]) and VMS score ≥1 (OR 2.9 [95% CI 1.1–7.9]) were positively associated with 30-day PCI-related SAE (Tab. 3).

**Table 3:** Univariable and multivariable logistic regression analysis of 30-day serious adverse events

	univariable logistic regression		multivariable logistic regression	
	OR (95% CI)	*p*-value	OR (95% CI)	*p*-value
*demographic*				
– age, years	1.1 (1.1–1.2)	0.000	1.1 (1.0–1.2)	0.017
– female	2.9 (1.3–6.5)	0.009	2.1 (0.8–5.4)	0.130
*risk factors*				
– treated hypertension^a^	1.1 (0.5–2.5)	0.853	–	–
– treated hyperlipidaemia^b^	1.0 (0.4–2.7)	0.936	–	–
– diabetes	3.6 (1.4–9.2)	0.009	1.4 (0.4–4.8)	0.590
– current smoker	1.0 (0.4–2.7)	0.957	–	–
– family history of CVD	0.9 (0.3–2.4)	0.805	–	–
*comorbid conditions*				
– history of cancer	0.7 (0.2–2.4)	0.526	–	–
– history of peripheral vascular disease	4.0 (1.2–13.4)	0.025	3.6 (0.9–15.3)	0.079
– history of cerebrovascular disease	1.7 (0.6–5.0)	0.315	–	–
– previous myocardial infarction	1.7 (0.6–4.8)	0.348	–	–
– previous PCI	0.9 (0.3–3.1)	0.927	–	–
– previous CABG	0.0 (0.0–)	0.999	–	–
*clinical characteristics*				
– out-of-hospital cardiac arrest	4.9 (1.0–23.2)	0.044	6.7 (1.0–43.1)	0.045
– anterior infarction	1.4 (0.6–3.1)	0.420	–	–
– abciximab administration	0.5 (0.2–1.3)	0.158	–	–
– VMS score ≥1	4.2 (1.9–9.7)	0.001	2.9 (1.1–7.9)	0.038

*CABG* coronary artery bypass graft surgery, *CI* confidence interval, *CVD* cardiovascular disease, *OR* odds ratio, *PCI* primary percutaneous coronary intervention, *VMS* Safety Management Programme

^a^Defined as systolic blood pressure ≥140 mm Hg and/or diastolic blood pressure ≥90 mm Hg and/or the use of antihypertensive medication

^b^Serum total cholesterol ≥6.0 mmol/l and/or serum triglycerides ≥2.2 mmol/l or treatment with lipid-lowering drugs

## Discussion

The key findings of this study are: (1) in patients older than 70 years treated with primary PCI for STEMI, a positive frailty score was associated with short-term mortality and short-term PCI-related SAE, most commonly 30-day death (5%) and major bleeding (9%); (2) these findings were independent of age and clinical characteristics. Patients with at least one sign of frailty had a nearly 10 times higher risk of 30-day mortality. The results of this study underline the fact that frailty in the elderly should be taken into account when developing risk models.

The number of elderly patients with STEMI referred for invasive treatment has increased in the past decade. It is widely reported that this patient group is at high risk for SAE and mortality [1–4]. For example, Antonsen et al. found that twice the number of octogenarians with STEMI were treated with primary PCI in 2009 in comparison with 2002 and that they had the highest short- and long-term mortality [5]. Advanced age, therefore, is a generally accepted risk factor for impaired outcomes in patients with STEMI. In addition to numerous studies that have found that high age is independently associated with both morbidity and mortality [6–10], established risk scores such as the GRACE risk score for patients with acute coronary syndromes (ACS) and the TIMI risk score for patients with STEMI incorporate age as a variable [19, 20]. However, the elderly population is heterogeneous, which emphasises the importance of further risk stratification.

Overall, it has been shown that frail patients are at risk for morbidity and mortality. It is estimated that 25–50% of the elderly above 85 years are frail, which makes it the most problematic expression of the aging population. Frailty is described as a state of vulnerability and impaired recovery of homeostasis after eliciting stressors, which can result in major changes in health status [11, 12]. The screening instruments for frailty used in this present study have been implemented nationwide as part of a mandatory programme since 2012 [21]. Heim et al. previously tested the feasibility and predictive performance of this VMS frailty score on 3‑month adverse outcomes in patients ≥70 years admitted to the hospital for acute conditions for at least 2 days. They found that the strongest predictive model for frailty was a positive score on ≥3 VMS domains if aged 70–80 years; or a positive score on ≥1 VMS domains if aged ≥80 years (sensitivity 68%, specificity 74%) [14].

Frailty as a risk factor for impaired clinical outcomes in elderly persons with myocardial infarction is a rather new concept. The predictive value of frailty has mainly been investigated in older patients with ACS, in most cases not requiring treatment with primary PCI. Ekerstad et al. were among the first to describe the independent association between frailty and both short- and long-term clinical outcomes in patients ≥75 years with non-ST segment elevation myocardial infarction (NSTEMI) [22, 23]. More recently, Alonso Salinas et al. found comparable associations in elderly patients with ACS [24]. Sujino et al. did address the issue particularly for patients with STEMI requiring primary PCI, and found similar results for this patient group [25]. The sample size of this study was, however, small (*n* = 62) and only patients ≥85 years were included. Another study conducted by Singh et al. found that the addition of frailty to a conventional risk score improved the prognostic power to predict long-term outcomes [26]. Patients that died in hospital were excluded, however, which makes these findings inapplicable to predict outcomes for all elderly patients referred for primary PCI. To our knowledge, this present study is the first to properly assess the relation between frailty and outcome in elderly patients with STEMI requiring primary PCI. The fact that we found that frailty, independent of age, is associated with short-term mortality and PCI-related SAE is important, since it enables more careful selection of high-risk groups than selection by age. For example, better selection of high-risk elderly persons by recording frailty in large registries such as the NHR could lead to specific improvement of care, such as involvement of geriatricians at an early stage of admission to prevent adverse events. In addition, including frailty next to age as a patient characteristic could result in an improved overview of regional differences between patient populations, which makes comparison of care in different hospitals more objective. Finally, if a high risk of short-term mortality after PCI in frail, elderly persons is confirmed by large registries, awareness could be raised that treatment preferences must be discussed by general practitioners, nursing home doctors or other specialists to avoid overtreatment.

The present analysis had several limitations. The study was observational and thus shares the limitations of all observational analyses. Since too many patients were lost to follow-up, no long-term follow-up data could be presented. Additionally, the study was underpowered to assess which features of frailty were most predictive for adverse events. Finally, study data were collected until 2015, which may limit the generalisability to the current elderly population despite the fact that no major differences in either the population or healthcare have occurred in the past 2 years. In the future, it could be evaluated which features of frailty are mostly associated with impaired outcomes in a larger cohort. The association between frailty and quality of life as an outcome measure might be of interest as well, to gain even more insight into the prognosis of the elderly STEMI patient.

## Conclusion

In conclusion, in this cohort of STEMI patients ≥70 years and treated with primary PCI, the 30-day survival was 95% and event-free survival was 77%. The presence of at least one feature of frailty was independently associated with both mortality as the combined endpoint of PCI-related SAE. These findings might enable more careful selection of high-risk groups in the heterogeneous group of elderly STEMI patients.

## Caption Electronic Supplementary Material

**Supplemental file 1. **Table 1 Baseline characteristics of patients with follow-up versus patients without follow-up for 30-days serious adverse events
