# Supplementary material for: Frailty score for elderly patients is associated with short-term clinical outcomes in patients with ST-segment elevated myocardial infarction treated with primary percutaneous coronary intervention
Source: Neth Heart J. 2019 Feb 15;27(3):127–33. doi: 10.1007/s12471-019-1240-7 (PMC6393578; doi:10.1007/s12471-019-1240-7)
Supplement: Supplementary file 1 — Supplemental file 1. Table 1 Baseline characteristics of patients with follow-up versus patients without follow-up for 30-days serious adverse events [file 12471_2019_1240_MOESM1_ESM.docx]

**Supplementary files**

***Supplemental file 1.*** *Table 1. Baseline characteristics of patients with follow-up versus patients without follow-up for 30-days serious adverse events*

|  | | **Follow-up 30-days serious adverse events** | | |
| --- | --- | --- | --- | --- |
|  | | **Yes** | **No** |  |
| Variable | | **n=143** | **n=63** | p-Value |
| Demographic | |  |  |  |
|  | Age, mean (SD), y | 78 (6,1) | 81 (6,7) | 0,008 |
|  | Female | 58 (41) | 29 (46) | 0,464 |
| Risk factors | |  |  |  |
|  | Treated hypertension† | 82 (60) | 42 (69) | 0,227 |
|  | Treated hyperlipidemia‡ | 31 (23) | 17 (28) | 0,443 |
|  | Diabetes | 23 (16) | 8 (13) | 0,593 |
|  | Current smoker | 28 (21) | 15 (26) | 0,427 |
|  | Family history of CVD | 31 (24) | 13 (22) | 0,809 |
| Comorbid conditions | |  |  |  |
|  | History of cancer | 18 (13) | 13 (21) | 0,142 |
|  | History of peripheral vascular disease | 12 (8) | 5 (8) | 0,902 |
|  | History of cerebrovascular disease | 19 (13) | 8 (13) | 0,926 |
|  | Previous myocardial infarction | 19 (13) | 12 (19) | 0,287 |
|  | Previous PCI | 18 (13) | 5 (8) | 0,346 |
|  | Previous CABG | 6 (4) | 3 (5) | 0,855 |
| Clinical characteristics | |  |  |  |
|  | Out of hospital cardiac arrest | 7 (5) | 2 (3) | 0,578 |
|  | Anterior infarction | 3 (2) | 1 (2) | 0,807 |
|  | Abciximab administration | 48 (34) | 22 (35) | 0,850 |
|  | VMS score ≥1 | 106 (75) | 45 (73) | 0,696 |

*Abbreviations: CABG, Coronary Artery Bypass Graft Surgery; CVD, Cardiovascular Disease; PCI, Primary Percutaneous Coronary Intervention; SD, Standard Deviation; VMS, Safety Management Program; Y, Year*

*† Defined as systolic blood pressure ≥140 mm Hg and/or diastolic blood pressure ≥90 mm Hg and/or the use of antihypertensive medication. ‡ Serum total cholesterol ≥6.0 mmol/L and/or serum TG ≥2.2 mmol/L or treatment with lipid lowering drugs.*
